# Improving Executive Functions at School in Children With Special Needs by Educational Robotics

**DOI:** 10.3389/fpsyg.2019.02813

**Published:** 2020-01-09

**Authors:** Maria Chiara Di Lieto, Emanuela Castro, Chiara Pecini, Emanuela Inguaggiato, Francesca Cecchi, Paolo Dario, Giovanni Cioni, Giuseppina Sgandurra

**Affiliations:** ^1^Department of Developmental Neuroscience, Istituto di Ricovero e Cura a Carattere Scientifico (IRCCS) Fondazione Stella Maris, Pisa, Italy; ^2^Department of Education, Language, Interculture and Psychology, University of Florence, Florence, Italy; ^3^The BioRobotics Institute, Scuola Superiore Sant’Anna, Pisa, Italy; ^4^Department of Clinical and Experimental Medicine, University of Pisa, Pisa, Italy

**Keywords:** educational robotics, special needs, response inhibition, working memory, executive functions, children

## Abstract

Children with Special Needs represent a highly heterogeneous group in terms of neurofunctional, behavioral, and socio-cognitive characteristics, but they have in common a frequent impairment of Executive Functions. Educational Robotics is generally dedicated to study the effects of constructing and programming robots based on children’s learning and academic achievement. Recently, we found that being engaged in progressively more challenging robot planning and monitoring (ER-Lab) promotes visual–spatial working memory and response inhibition in early childhood during typical development, and that an ER-Lab can be a feasible rehabilitative tool for children with Special Needs. The present study aimed to verify the efficacy of the ER-Lab on Executive Functions in children with Special Needs for the first time by using an RCT within their school environment. To pursue these aims, this study reports the results obtained in 42 first-grade children with Special Needs engaged in school Educational Robotics Laboratories (ER-Lab) to promote Executive Functions by means of enjoyable, intensive, and incrementally more challenging activities requiring them to program a bee-shaped robot called Bee-bot^®^ (Campus Store). Several adaptations were done to meet different motor, cognitive, and social needs. All children were evaluated by means of standardized tests performed by each child before and at the end of the ER-Lab activities. Children with Special Needs had significantly improved inhibition skills, and children with attentional impairment had more benefits in their inhibition of motor responses tasks with respect to children with a language deficit. Results of the study and future perspectives on how ER-Lab programs could become a powerful tool in classrooms with children with special needs are discussed.

## Introduction

Children with Special Needs (SN) require exceptional educational and teaching strategies because of social, physical, or mental problems. They represent a highly heterogeneous group in terms of neurofunctional, behavioral, and socio-cognitive features. Children with SN may have sensorial or motor disabilities, Autism Spectrum Disorders, Mild or Severe Intellectual Disabilities, and specific neurodevelopmental disorders, such as Attention Deficit Hyperactivity Disorder (ADHD), Specific Learning Disorders, Specific Language Disorders, or other unspecified difficulties (McFarland et al., 2018; MIUR – Ufficio Statistica e Studi, 2018). Despite this variability, it is nowadays well accepted that specific processes for cognitive control, such as Executive Functions (EFs), are frequently impaired across different developmental disorders and special needs (Pennington and Ozonoff, 1996). EFs have been found to be frequently altered in children with socio-economic disadvantages (Noble et al., 2007), Mood Disorders (Vilgis et al., 2015), Attention Deficit Hyperactivity Disorder (ADHD) (Castellanos et al., 2006), Autistic Spectrum Disorder (ASD) (Pellicano, 2012; Margari et al., 2016), Language and Learning Disabilities (Moll et al., 2014; Kapa and Plante, 2015; Peng and Fuchs, 2016), Down Syndrome (DS) (Lott and Dierssen, 2010; Lanfranchi et al., 2015), neuromuscular disorders (Astrea et al., 2016; Battini et al., 2018), and Cerebral Palsy (CP) (Pirila et al., 2011; Di Lieto et al., 2017a). The casual relationship between EF impairment and Special Needs is far from linear as three main scenarios may be suggested: in some circumstances, a clear EF deficit is a part of the “core cognitive difficulties” of a certain SN group; in other conditions, only subtle difficulties are found; finally, it may be that it is the clinical or social problem itself that induces the EF impairment (Astrea et al., 2016).

The complexity of the EFs–SN relationship may, in part, be due to the fact that EFs are a complex construct, described by different theoretical frameworks. Although multi-componential models define the main basic EF components differently (e.g., Miyake et al., 2000; Diamond, 2013; Friedman and Miyake, 2017; Morra et al., 2018), within a developmental prospective focused on early ages, there is agreement on their role as preciouses “tools of learning” for academic skills at different grades (Diamond, 2013). The ability to manipulate information held in the memory is highly involved in language acquisition, decoding, text comprehension (Swanson et al., 2009; Christopher et al., 2012), and in mathematical achievement, such as counting and mental arithmetic (St Clair-Thompson and Gathercole, 2006; Mammarella et al., 2010; Caviola et al., 2012; Viterbori et al., 2015). The ability to inhibit prepotent responses, concerning the suppression of compelling thoughts or memories and behavior, and resist distractor interference, which is selectively attuned to what we choose, thereby removing attention to other interferent stimuli, allows us to focus on relevant information during reading comprehension (Borella et al., 2010) or solving arithmetic problems (D’Amico and Passolunghi, 2009; Gilmore et al., 2015). Finally, the ability to rapidly change task, operations, mental sets, or strategies seems to be connected to academic learning (Bull and Lee, 2014). According to Diamond’s model (Diamond, 2013), these processes concern three main basic EFs components, namely working memory, inhibition, and cognitive flexibility. Inhibition, working memory, and, to a lesser extent, cognitive flexibility have frequently been found to be impaired in several types of Special Needs (Vicari and Di Vara, 2017).

Given the predictive role EFs have on academic achievement, early interventions on working memory and inhibition in children with SN may prevent cascade effects on quality of life, school attendance, and social functioning (Diamond and Lee, 2011).

Different approaches have been proposed to empower the main EF components in typical and atypical development. In the preschoolers, they have been focused mainly on self-regulation by paper and pencil school activities (Dias and Seabra, 2015; Traverso et al., 2015; Duncan et al., 2018; Howard et al., 2018; Diamond et al., 2019), while computerized training has been proposed mainly for school-aged children (Klingberg et al., 2005; Aksayli et al., 2019). Moreover, aerobics, martial arts, yoga, and mindfulness have recently been suggested as efficacious tools to empower EFs (Diamond and Lee, 2011). Results across the different studies are variable and not easily comparable because of theoretical and methodological differences. Among all, studies varied for the outcome measures used, for the generalization effects found, and for their conformity to different EF constructs (Morra et al., 2018; Aksayli et al., 2019). By reviewing the different approaches, Diamond and Lee (2011) suggested that, in order to empower the efficacy of the EF interventions and the power of generalization to several daily life activities, the presence of the following principles are needed: (i) constantly challenging activities (Diamond and Ling, 2016); (ii) adaptive and intensive schedules (Klingberg et al., 2005; Thorell et al., 2009); (iii) repeated practice (Diamond and Lee, 2011); (iv) the involvement of emotional, physical, and social aspects (Diamond and Lee, 2011); (v) variability of the tasks (Klingberg et al., 2005; Rueda et al., 2005; Wass et al., 2011); and (vi) the high-motivation mentoring skills of the trainers (Diamond and Ling, 2016).

In order to propose new EF training that embeds the above characteristics, the use of new technologies in day-to-day life and social contexts, such as school, may be promising.

Among the new technologies implemented for educational purposes, Educational Robotics (ER) has been used with typically developed children in educational settings to enhance problem solving, planning, and computational thinking (La Paglia et al., 2011; Benitti, 2012; Kazakoff and Bers, 2014), basic EFs components (Di Lieto et al., 2017b), and academical learning, especially in the area of Science, Technology, Engineering, and Mathematics (STEM area; Hussain et al., 2006; Barker and Ansorge, 2007; Nugent et al., 2008). ER refers to a learning approach based on the design, assembly, and programming of robots and takes its psycho-pedagogical background both from the constructivism and constructionism theories of learning and cognitive development (Piaget and Inhelder, 1966; Papert and Harel, 1991) and from social learning theories (Bandura, 1962; Bandura et al., 1966; Vygotsky, 1987).

Recently, an increasing number of studies have proposed ER to SN populations with the aim of offering new learning and socially inclusive opportunities. Examples of the application of robots, in both clinical and school settings, have been documented in different types of special needs (Cook et al., 2010; Cheng et al., 2018), including learning difficulties (Conchinha et al., 2016), motor disorders (Robins et al., 2012), intellectual disabilities (Businaro et al., 2014; Bargagna et al., 2018), autism (Robins et al., 2004; Robins et al., 2005), and ADHD (Fridin and Yaakobi, 2011).

Indeed, aside from elicit engagement and social behaviors (Diehl et al., 2011; Scassellati et al., 2012), STEM learning (Lindsay and Hounsell, 2017), play and exploration activities (Cook et al., 2000), educational robots have been used in the SN population to investigate specific cognitive functions, such as cognitive flexibility in children with ASD (Costescu et al., 2015) or the effect of robot-mediated learning (Krishnaswamy et al., 2014). The study by Krishnaswamy investigated the effects of a robotic training to improve visual motor skills in children with learning disabilities and visual motor delays, by comparing robot programming with traditional occupational therapy. The results showed that the children who participated in the ER activities improved visual–motor performances more than children following the traditional curriculum. Another study by Conchinha presented two single cases who, by participating in ER activities with Lego Mindstorm, improved learning, language, and inclusion (Conchinha et al., 2016). Finally, after finding that intense, challenging, and entertaining ER training (ER-Lab), organized according to incremental difficulty, improved visuo-spatial working memory and inhibition in typical preschoolers (Di Lieto et al., 2017b), we verified the feasibility of the ER-Lab in a group of children with Down Syndrome in a clinical setting (Bargagna et al., 2018).

The above evidence indicates that the ER-Lab is a flexible tool, adaptable to both clinical and educational environments for both SN and typically developing children, for cognitive improvement; indeed, it may be useful for personalizing interventions in neurodevelopmental disorders. The ER-Lab appears to simultaneously incorporate several characteristics to promote efficacy of the EFs trainings. ER-Lab activities may be intense, challenging, and adaptable to individual functioning, thus acting in the proximal development zone (Vygotsky, 1987); it can promote several EF components, either simultaneously or separately, because robot programming requires sequential reasoning before acting by inhibiting impulsive responses, holding and manipulating visuo-spatial and verbal information in memory, and shifting between different commands/rules (Di Lieto, submitted). ER activities can be performed in every school context, creating a group setting and an attractive learning environment, thus promoting students’ interest and motivation (Alimisis, 2013), and this allows for interventions not only on cognitive empowerment but also on social and emotional inclusion. Finally, the ER-Lab ensures the presence of a mentor who can adapt the activity to the need of the single subject.

Given the prevalence of the executive and visuo-spatial domains in the ER-Lab, our previous results (Di Lieto et al., 2017b) and in line with the recent theories of EFs development, which hypothesize a two-factor model with inhibition as a distinct dimension from working memory in children aged 5–7 years old (Usai et al., 2014), significant improvements in inhibition and visuo-spatial working memory were expected in first-grade children. In the present study, the ER-Lab was used in SN children with multiple aims:

• to evaluate the feasibility of an intensive school ER-Lab for children with SN in the first class of the primary school,
• to adapt the ER-Lab training to different types of SN children,
• to measure by standard tests of inhibition and visuo-spatial working memory the training effect of the ER-Lab in SN children,
• to compare the efficacy of the ER-Lab across SN subgroups differing for type and degree of the neuropsychological impairment,
• to estimate the improvements in the Bee-bot programming skills during the ER-Lab in SN children.

## Materials and Methods

### Participants

A total of 13 classes from nine schools participated in the study, from which 187 children with typical development and 42 children with SN from such classes (in Italy all children with SN attend regular classes) were selected (14 females; 28 males; age range 5–7 years, mean age 5.9; and standard deviation 0.7). To fulfill the goals of this study, only data collected from children with SN, identified on the basis of their medical certificates and on the basis of teachers’ reports, were presented and discussed. The phase of enrollment of the participants’ schools has been developed with the collaboration of the District of Pisa in order to reach as many schools as possible. This research project has been approved by the Pediatric Ethics Committee of Tuscany Region. All parents gave written consent for their children participating in the study and for the publication of the results.

### ER-Lab Training

The ER-Lab was conducted twice a week for 10 weeks (20 ER training sessions of 60 min) and involved not only the children with SN but all the children of the class. The ER-Lab was conducted during school time. To choose the most proper robot for our research purposes, a survey was conducted, individuating two models: Bee-bot (Campus Store), a bee-shaped robot, and Pro-bot (Campus Store), a car-shaped robot. Bee-bot robot was selected because it is one of the most utilized robots for school-aged children (Janka, 2008) as it is considered one of the most suitable hardwares for lower primary school children in educational technology (Janka, 2008), and it was expected to be challenging for children with SN aged 5–7.

**FIGURE 1:**
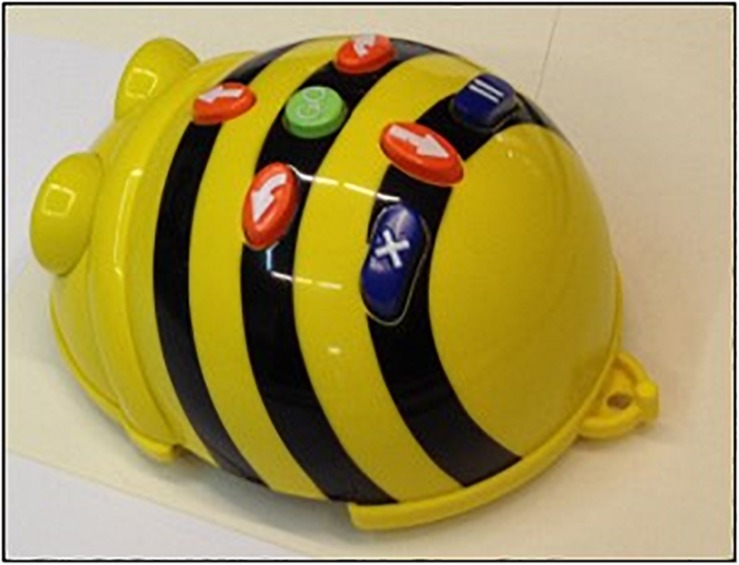
The Bee-bot robot.

Bee-bot has a child-friendly design, with a black/yellow bee outline (see Figure 1). The Bee-bot can be programmed by some buttons positioned on its back that allow the motion or the rotation of the robot. By four orange buttons it is possible to move the robot either forward or backward (15 cm), and rotate it right or left (90° rotation); a central green button (GO button) makes the programmed sequence start; a blue button removes memory of the robot and starts a new sequence that does not include the program previously inserted (CLEAR or X); another blue button programs a short stop during robot motion (PAUSE or II). At the end of the programmed sequence, Bee-bot furnishes visual and acoustic feedback.

**FIGURE 2:**
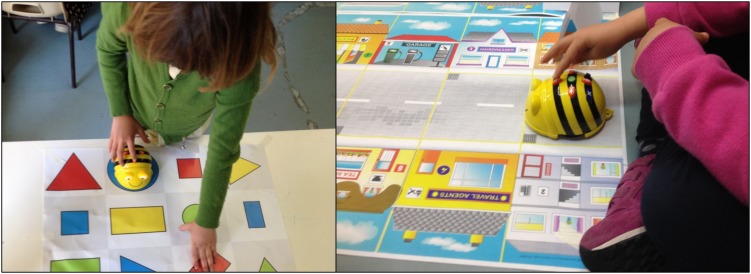
The carpets utilized with the Bee-bot robot.

During ER-Lab activity, specific activities were proposed, such as asking the child to move the Bee-bot robot in the space, delimited by a carpet (see Figure 2) representing a city map or another narrative context, to reach a specific area.

The ER-Lab activities were carried out in a group setting, dividing the children into small groups of five or six children maximum. This choice was made in order to promote the involvement of all the children favoring the observational learning, collaboration, and involvement among peers. Two teachers and one experimenter directed the ER-Lab in each class. According to an adaptive paradigm, the cognitive and robot-programming goals were progressively increased in terms of difficulty. To think before taking action was encouraged, promoting not only a “learn by doing” but also a “learn by thinking” approach and utilizing a metacognitive method.

Every week, the ER-lab trained specific cognitive competencies, focusing mainly on visuo-spatial working memory, response inhibition, and interference control. Mental planning, the capacity to rapidly switch mental sets or strategies during tasks (such as set-shifting and task-switching), language comprehension, and sustained attention were required too. The first 2 weeks were focused on robot familiarization thought simple visuo-spatial robot planning; the third and fourth weeks concentrated on the training of spatial working memory through the programming of more complex robot visuo-spatial planning; the fifth and sixth weeks were focused on robot activities that stressed working memory and inhibition abilities; the seventh and eighth weeks were focused on inhibiting automatic answers in set-shifting or task-switching robot tasks; and the ninth and tenth weeks were dedicated to improving academic skills through the use of robotic programming. Moreover, additional and optional activities, directed to the consolidation of the objectives, were included. Details of cognitive and robot-programming goals for children with SN and examples of adapted activities provided for each ER-Lab week are reported in Table 1.

**TABLE 1:** Details of cognitive and robot programming goals and example of activities and adaptations for each ER-Lab week.

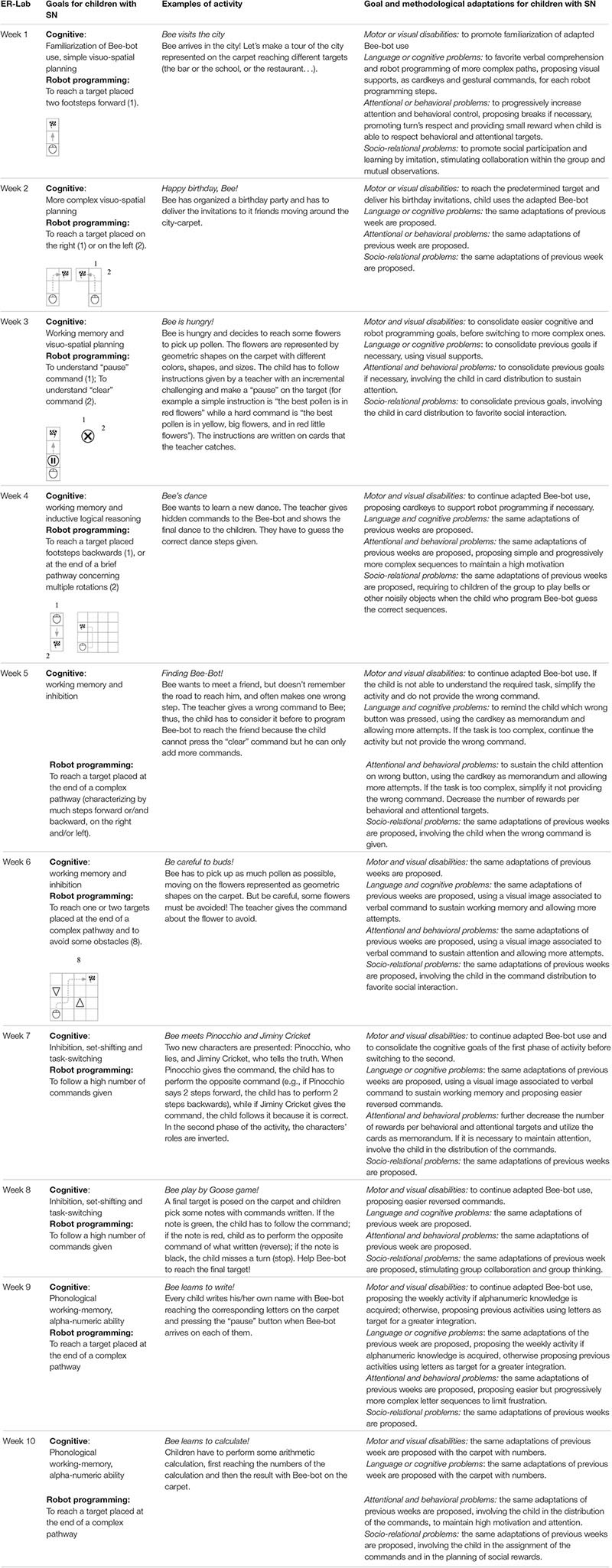

For SN children, *ad hoc* adaptations of both the robots and of the activities were proposed. General indications to perform the activities with SN children were followed. In particular:

• to work in a small group,
• to place the child near the teacher and in a place with few distractions,
• to favor the teamwork and collaboration between children,
• to favor attention and motivation toward customizable reinforcements.

Examples of adaptation of the activities are the following:

• For children with linguistic or cognitive problems, some cardkeys were created, representing the different buttons of Bee-bot. The cardkeys helped the children in the robot programming by being a visual prompt to be associated with the oral command in order to facilitate the learning and permitting a non-verbal response in case of linguistic problems.
• For children with attentional and behavioral problems, attention time was progressively increased, frequent breaks were proposed, and token economy strategies were used to introduce the respect of the group activity rules, such as the turn respect.
• For children with socio-relational problems, imitation learning, collaboration, and involvement among peers were favored throughout relational reinforcements.

**FIGURE 3:**
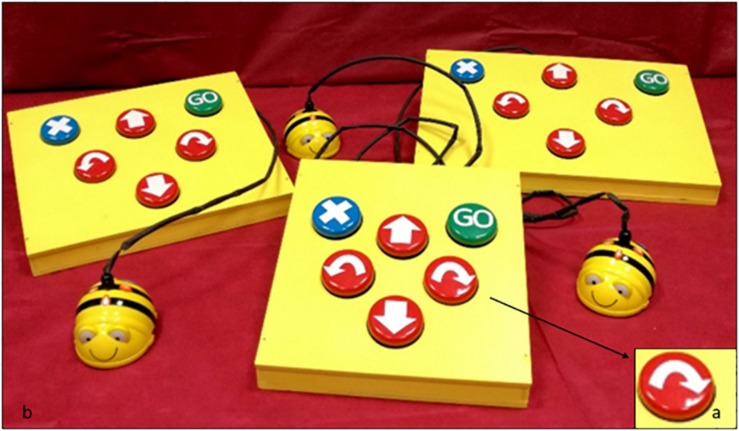
**(a)** Switched on/off sensors of 65 mm diameter, Jelly Bean; **(b)** The adapted Bee-bot.

In addition to this, Bee-bot has been adapted to children with motor or visual disabilities who could have had difficulties in using small commands to program Bee-bot. Thus, the programming interface was modified, and special larger sensors, switched on/off sensors of 65 mm diameter (Jelly Bean), were inserted in the place of the original ones (Figure 3). Modified Bee-bot was used for children with cognitive disability too as Jelly Bean sensors could be temporarily put off-line, thus limiting the choices of planning and making the activities simpler.

### Study Design

**FIGURE 4:**
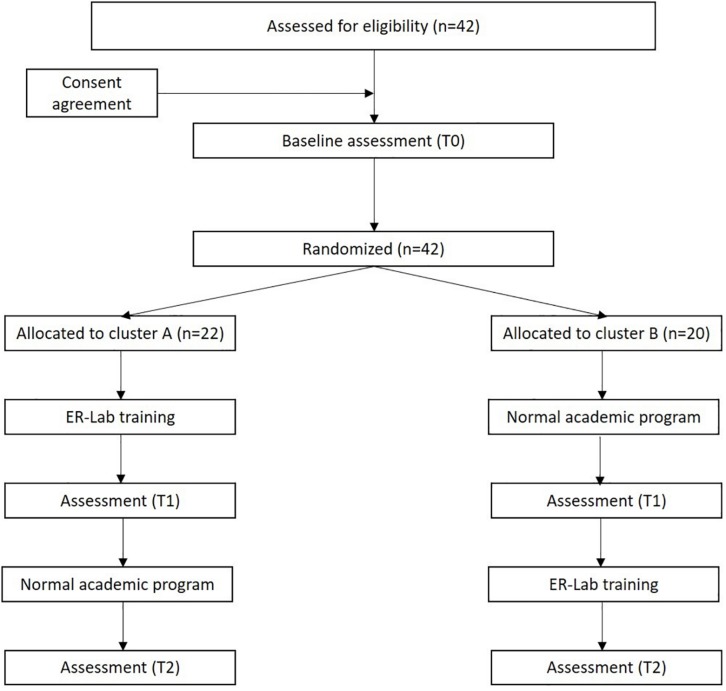
The study flow diagram.

According to the waitlist randomized trial design, the school classes were randomly split into two groups, and children with SN were thus divided in two Experimental Conditions (Experimental Condition A, *n* = 22 and Experimental Condition B, *n* = 20) for the sequential training rollout. Given this study design, children with a diverse degree and type of impairment were not evenly distributed in the groups under the two conditions. Both experimental conditions were assessed by neuropsychological tests (for details see section Outcome Measures) at time point T0 (in September 2016). After the evaluation, children in Experimental Condition A immediately started ER-Lab training, while those in Experimental Condition B continued their normal academic program. After 10 weeks, all children (Experimental Condition A and B) were re-tested at time point T1 (January 2017). After T1 assessment, Experimental Condition B started ER-Lab training, while Experimental Condition A continued normally academic program. After another 10 weeks, all children were retested at time point T2 (May 2017) (see Figure 4 for the Study Flow Diagram). The evaluators, who tested children at the three time points, recorded the data, while separate examiners collected and entered data in a database. The evaluators and examiners were blind to the study design and external to the research team.

### Outcome Measures

In order to accomplish the aims mentioned, several tests tapping into visuo-spatial working memory and inhibition were selected. Children were assessed by standardized neuropsychological tests and qualitative measures of robotic-programming skills. Several tests were used.

#### Visuo-Spatial Memory

• Forward Corsi Block Tapping subtest (BVS test). This test measured visuo-spatial memory through the evaluation of the span, representing the longest visuo-spatial information sequence that the child could remember. The visuo-spatial sequence was represented by a sequence of blocks, inserted in a plastic board, that the child had to touch in the same order that the examiner did. The longest sequence of blocks correctly repeated represented the span obtained, and this was computed as the final score of the test (range score 2–8) (Mammarella et al., 2008).

#### Executive Functions

##### Visuo-spatial working memory

• Backward Corsi Block Tapping subtest (BVN test). This test was similar to the Forward Corsi Block Tapping subtest, but it measured visuo-spatial working memory by asking the child not only to remember but also to manipulate visuo-spatial information by touching the blocks indicated by the examiner in the reverse order. The longest sequence of blocks correctly repeated in the reverse order represents the span backward obtained, and it was then computed as the final score of the test (range score 1–7) (Bisiacchi et al., 2005).
• Matrix Paths (BVS-Corsi). This test assessed verbal and visuo-spatial working memory by asking the child to identify the final destination on a matrix by listening to a sequence of spatial steps read by the examiner that got progressively longer. The final score was the sum of the correct responses (range score 0–30) (Mammarella et al., 2008).

##### Prepotent response inhibition and interference control

• Inhibition subtest (NEPSY-II test). This test measured the ability to inhibit automatic verbal answers in favor of no-intuitive ones. The first condition was the baseline (Naming condition). The child had to denominate a sequence of alternating figures (square and circle). In the second condition, the Inhibition one, the child had to name “circle” when a square was present and to name “square” when a circle was present. In this test, the score was made by computing the number of errors (range score 0–40), self-correcting responses (range score 0–40), and time (range score 0”–240”) of both conditions. All the scores were included in the statistical analysis (Korkman et al., 2007; Urgesi and Fabbro, 2011).
• Little frog’s subtest (BIA). This test assesses sustained attention and the ability to inhibit automatic motor answers. The child had to listen to a sequence of acoustic commands: a “Go” command, which indicated that the child should make a graphic tick with a pencil, and a “No-Go” command, very similarly to the first one, which indicated that the child should stop the graphic sequence. The number of correct responses were counted (range score 0–20) (Marzocchi et al., 2010).
• Pippo-says test (a modified version of Simon-says). This test mainly assessed motor inhibition. In this test, two conditions were present: in the first one, the examiner read a sequence of commands to the child that he had to perform only if the command started with the words “Pippo dice.” In the second condition, the one utilized in the present study, the instructions were identical, but the examiner performed all the command, and so the child had to inhibit the command not starting with “Pippo dice,” and at the same time, control the interference due to examiner performances. Each condition as made by 10 commands. The number of correct commands were computed (0–10 range score) (Marshall and Drew, 2014).

#### ER-Lab Test

**FIGURE 5:**
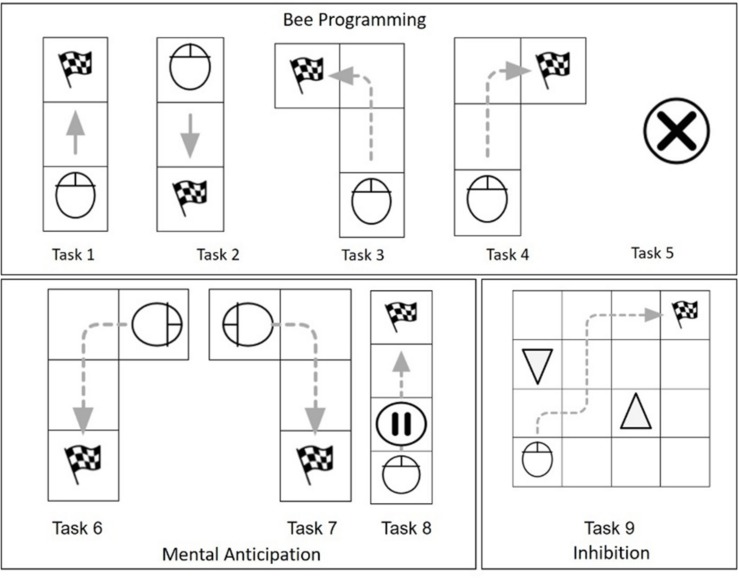
The ER-Lab test.

In our first pilot study (Di Lieto et al., 2017b), an ER-Lab test was created to estimate the improvements in the Bee-bot programming skills during the ER-Labs. The test was composed of nine tasks, and they were divided into subscales on the basis of their complexity: (i) tasks one to five assessed Bee-bot simple utilization (Bee Programming); (ii) tasks six to eight assessed the ability to plan complex visuo-spatial pathways (Mental Anticipation); (iii) task nine assessed inhibition abilities during Bee-bot navigation (Inhibition) (Figure 5).

The ER-Lab test was administered at the beginning, at the middle, and at the end of ER-Lab training. Zero points were accredited if the goal was not reached, half a point was given if the goal was achieved with concrete support (such as anticipating correct navigation by using their own hand or the Bee-bot), and one point was given if the goal was reached without any concrete help.

#### ER-Lab Logbooks

At the end of each week, teachers filled a logbook in which different aspect of the ER-Lab were qualitatively evaluated. In particular, teachers were asked to report the principal weakness and strengths of children that were met during the ER-Lab training activities of the week.

### Statistical Analysis

All statistical analyses were performed using R, the R Project for Statistical Computing software package, version 3.6.0, with a significance level of 5%.

Given the high heterogeneity of the sample, preliminary analysis of the pre-training assessment was conducted based on the degree (mild vs. severe) and the type (attention vs. language problem) of impairment by independent sample Student t tests in case normality assumptions were met. Mann–Whitney tests were used otherwise.

In order to test the effect of the training, separate linear mixed-effects models for each outcome measure were used, with (binary) variables representing ER-Lab training and Experimental Condition A/B as fixed factors and subject ID as random factor, in a repeated measure design. Family-wise estimations obtained by general linear hypotheses were used to test for the following two *post hoc* contrast variables of interest in determining neuropsychological differences during ER-Lab training in both Experimental Conditions (names assigned are indicative of interpretation of the contrasts):

• Training Effect. This was calculated by adding delta changes for time points T1 and T0 for Experimental Condition A and delta changes for time points T2 and T1 for Experimental Condition B.
• Within Baseline Effect. This was calculated by adding delta changes baseline in Experimental Condition B (T1–T0 for Experimental Condition B) and follow-up in Experimental Condition A (T2–T1 for Experimental Condition A).

The differences in the training effects according to the degree and type of impairments were evaluated, comparing pre-post delta changes in each neuropsychological outcome measure between subgroups.

Repeated measure ANOVAs, with *post hoc* Bonferroni corrections to *p*-values, were performed to test differences in ER-Lab tests at the beginning, middle, and end sessions of the training.

## Results

### Sample Characteristics

Clinical and descriptive data of the sample are listed in Table 2.

**TABLE 2:** Clinical and descriptive data of the study group of children with SN.

	**Age range (y,m)**	**Schools**	**Classes**	**Type of cognitive or neuropsychological impairment**	**Degree of impairment**
**Experimental condition A**					
S1	6,51–7,00	School 1	Class 1	Language	Mild
S2	6.51–7,00	School 1	Class 1	Attention	Mild
S3	5,51–6,00	School 1	Class 1	Cognitive impairment	Mild
S4	6,01–6,50	School 1	Class 1	Attention	Mild
S5	5,01–5,50	School 1	Class 1	Attention	Mild
S6	5,51–6,00	School 2	Class 2	Cognitive impairment	Mild
S7	5,51–6,00	School 2	Class 2	Attention	Mild
S8	5,51–6,00	School 2	Class 2	Language	Mild
S9	7,51–8,00	School 2	Class 2	Attention	Mild
S10	8,01–8,50	School 2	Class 2	Language	Mild
S11	5,51–6,00	School 3	Class 3	Intellectual deficit	Severe
S12	5,51–6,00	School 3	Class 4	Autism	Severe
S13	5,01–5,50	School 3	Class 3	Cognitive impairment	Mild
S14	5,01–5,50	School 3	Class 3	Attention	Mild
S15	5,01–5,50	School 3	Class 3	Attention	Mild
S16	5,01–5,50	School 3	Class 4	Cognitive impairment	Mild
S17	5,01–5,50	School 4	Class 5	Language	Mild
S18	6,01–6,50	School 4	Class 5	Cognitive impairment	Mild
S19	7,51–8,00	School 5	Class 6	Intellectual deficit	Severe
S20	5,51–6,00	School 5	Class 6	Attention	Mild
Mean (*SD*)	6,90 (0,9)				
Range	5,01–8,00				
**Experimental condition B**					
S21	6,01–6,50	School 6	Class 7	Cognitive impairment	Severe
S22	7,01–7,50	School 6	Class 7	Motor disorder	Severe
S23	7,01–7,50	School 6	Class 7	Attention	Severe
S24	6,01–6,50	School 6	Class 7	Autism	Severe
S25	6,01–6,50	School 6	Class 7	Language	Mild
S26	6,01–6,50	School 6	Class 7	Language	Mild
S27	5,51–6,00	School 6	Class 7	Attention	Mild
S28	6,51–7,00	School 6	Class 7	Cognitive impairment	Mild
S29	7,51–8,00	School 7	Class 8	Intellectual deficit	Severe
S30	6,51–7,00	School 7	Class 8	Intellectual deficit	Severe
S31	6,51–7,00	School 7	Class 9	Motor disorder	Severe
S32	6,01–6,50	School 7	Class 9	Attention	Mild
S33	5,51–6,00	School 8	Class 10	Cognitive impairment	Mild
S34	6,01–6,50	School 8	Class 10	Attention	Mild
S35	6,01–6,50	School 9	Class 11	Motor disorder	Severe
S36	7,01–7,50	School 9	Class 12	Intellectual deficit	Severe
S37	6,01–6,50	School 9	Class 13	Cognitive impairment	Severe
S38	5,51–6,00	School 9	Class 11	Cognitive impairment	Mild
S39	5,01–5,50	School 9	Class 11	Attention	Mild
S40	5,01–5,50	School 9	Class 13	Attention	Mild
S41	5,51–6,00	School 9	Class 13	Language	Mild
S42	6,01–6,50	School 9	Class 13	Language	Mild
Mean (*SD*)	6,4 (0,6)				
Range	5,01–8,00				

Children showed different special needs: 14 had attentional problems, 8 had language difficulties, 10 had cognitive impairment, 5 had intellectual deficits, 2 had Autism Spectrum disorder, and 3 had neuro-motor disabilities. The degree of the impairment varied across children: 13 out of 42 children had more severe clinical problems and needed Learning Support Teachers in their classroom who provided them help to reach maximum proficiency in academic achievements for their possibilities, while 29 children showed minor clinical impairments and pursued the academic objectives of their classes using methodological adaptations based on their specific clinical impairments (see Table 2).

Comparing children according to the degree of the impairment, reported in Table 2, differences at pre-training assessment were only found in the Forward Corsi Block Tapping test [*t*(38) = -2.07, *p* = 0.045] as children with minor clinical problems showed better performances when compared to those with severe clinical impairment.

Concerning clinical subgroups, which were divided according to the type of neuropsychological impairment, for two of them (Autism Spectrum Disorder and Intellectual Disability), no outcome measures were administrable due to the strict rules of standardized measures to obtain reliable data. Moreover, because of the small sample size and the high internal variability of other neuropsychological subgroups, it was not possible to directly compare all the different subgroups. For a visual inspection of data see Table 3.

**TABLE 3:** Mean and Standard Deviation on pre- and post-training performances for each outcome in each neuropsychological subgroup.

**Subgroups according the type of neuropsychological impairment**	**Pre- or post- training**	**Forward Corsi Block Tapping test**	**Backward Corsi Block Tapping test**	**Matrices Paths test**	**Time in naming condition**	**Errors in naming condition**	**Self-correcting responses in naming condition**	**Time in inhibition condition**	**Errors in inhibition condition**	**Self-correcting responses in inhibition condition**	**Little frogs test**	**Pippo says test**
Attentional	Pre-	3.14 ± 1.03	2.00 ± 0.78	6.07 ± 4.14	94.43 ± 29.74	2.79 ± 6.22	2.50 ± 2.98	110.36 ± 33.46	5.64 ± 8.97	6.00 ± 5.38	9.14 ± 2.93	6.21 ± 1.97
problems	Post-	3.36 ± 0.74	2.21 ± 1.05	8.14 ± 3.68	68.93 ± 13.63	1.36 ± 1.78	1.43 ± 1.02	99.78 ± 27.13	4.71 ± 6.30	4.43 ± 3.06	11.36 ± 5.24	7.71 ± 1.90
Language	Pre-	2.88 ± 0.83	2.00 ± 1.07	2.43 ± 2.70	107.43 ± 54.04	1.86 ± 2.11	2.14 ± 1.34	149.14 ± 72.68	6.86 ± 7.69	3.71 ± 2.81	7.00 ± 3.51	7.14 ± 2.79
difficulties	Post-	3.50 ± 0.53	2.25 ± 1.03	6.14 ± 4.22	90.57 ± 41.67	1.14 ± 1.46	1.57 ± 1.27	127.43 ± 57.66	6.43 ± 7.85	4.00 ± 3.27	7.57 ± 5.91	6.14 ± 1.46
Cognitive	Pre-	2.70 ± 0.67	1.70 ± 0.48	2.70 ± 1.89	104.40 ± 41.16	2.40 ± 3.24	3.70 ± 3.71	132.30 ± 45.45	12.30 ± 14.88	5.10 ± 3.41	6.70 ± 7.10	5.60 ± 2.84
impairment	Post-	2.80 ± 0.42	2.20 ± 0.92	4.80 ± 2.97	88.20 ± 32.79	3.00 ± 4.03	1.50 ± 1.65	123.20 ± 48.49	8.10 ± 8.77	4.80 ± 3.29	8.10 ± 5.72	7.00 ± 2.62
Neuromotor	Pre-	2.67 ± 0.58	2.00 ± 1.00	6.00 ± 3.46	132.67 ± 32.02	3.00 ± 2.65	8.00 ± 8.66	159.67 ± 6.66	7.67 ± 6.43	7.00 ± 6.08	5.67 ± 4.16	8.33 ± 2.89
disabilities	Post-	2.33 ± 0.57	2.33 ± 0.58	7.67 ± 0.57	84.67 ± 16.50	1.33 ± 0.58	1.67 ± 0.58	123.33 ± 20.50	4.33 ± 1.53	4.67 ± 6.43	3.33 ± 1.52	6.67 ± 3.05

**NS, not available.**

For this reason, statistical analyses were run to compare children with attentional (*n* = 14) and language (*n* = 8) problems in order to verify whether difficulties in sustaining attention or in instruction comprehension could affect the ER-Lab efficacy. At pre-training assessment, children with language problems showed significant worse performances in the Matrices Path tests [*t*(20) = 2.28, *p* = 0.033] compared to the other subgroup, and no other difference between these two subgroups was found at pre-training assessment.

### Outputs of Feasibility Study

#### The Small Group Context

From the qualitative analysis of the ER-Lab logbooks, all children performed the ER-Lab within a small group setting, showing motivation and interest in proposed activities and in social interactions with other children. Only one child (S36) had not followed activities in a group context due to the severe cognitive, motor, and visual problems, which required a one-to-one relationship with the teacher. However, this child performed the ER-Lab sessions within the classroom, and could thus observe the performances of other children and obtain encouragement and incentive from the others.

#### Methodological and Goals Adaptations

Children with attentional impairments carried out frequent breaks to maintain high levels of motivation and better focus on behavioral control and on activities. The token economy strategy had been performed only with children with hyperactivity disorders in addition to attentional problems. Cardkeys had been used with children, both with those with verbal comprehension deficits and those with intellectual disabilities, to facilitate and decompose the different robot steps needed for the more complex sequences of planning. For children with autism, the ER-Lab activities were planned in smaller groups of children, beginning with a one-to-one activity, mediated by an adult, and progressively inserting the child with autism into bigger groups of children in order to promote imitation learning, collaboration, and social involvement, adaptations particularly crucial for children with autism.

The modified Bee-bots had been proposed to children with motor disorders, intellectual disability (S36, S30), and with autism. The children with motor disorders did not show an interest or perceived benefit from this adaptation because the motor problems concerned inferior limbs or one side of the body. Only one of these children (S36) had continually used the modified Bee-bot, showing motivation and pleasure. The other child with a severe intellectual disability and a child with autism, instead, preferred to use modified Bee-bot as an alternative to the standard Bee-bot in order to feel more integrated in the activities.

The adaptations of the robot programming request and of the cognitive goals were used with all children with intellectual disabilities who needed to repeat the same activities several times, also in subsequent sessions, to reach minimum goals.

#### Neuropsychological Assessments

Three children with more severe problems (S19, S29, and S24) did not complete all tests at all time points. Only one child (S36) did not perform any test because of the severity of the difficulties, thus he was excluded from statistical analysis. Not relevant difficulties were found in the neuropsychological assessments of the other children.

### Effect of the ER-Lab Training

Comparing Experimental Conditions, no difference in chronological age [*t*(40) = −1.7, ns], gender [χ^2^(1) = 0.05, ns], or any neuropsychological tests at T0 time points (*p >* 0.05) were found.

As shown in Table 4, at the end of the training, improvement performances were found in 54% of children in the Matrices Path test, in 77 and 66% of children in the Naming and Inhibition speed, and in 55% of children in the Inhibition self-correcting responses.

**TABLE 4:** Descriptive data on pre- and post-training performances for each outcome in children with SN.

**Neuropsychological outcomes**	**Pre-training^∗^ Mean ± SD**	**Post-training° Mean ± SD**	**% of children with improve performances^+^**
Forward Corsi Block Tapping test	2.80 ± 0.85	2.95 ± 0.80	32%
Backward Corsi Block Tapping test	1.85 ± 0.77	2.13 ± 0.96	33%
Matrices Paths test	3.83 ± 3.56	5.90 ± 4.00	54%
Time in naming condition	104.97 ± 38.26	87.20 ± 36.08	77%
Errors in naming condition	2.45 ± 4.18	2.02 ± 2.93	44%
Self-correcting responses in naming condition	3.13 ± 3.67	1.69 ± 1.42	49%
Time in inhibition condition	129.82 ± 47.34	116.26 ± 41.37	66%
Errors in inhibition condition	8.55 ± 10.27	7.24 ± 8.93	50%
Self-correcting responses in inhibition condition	5.00 ± 4.33	4.24 ± 3.29	55%
Little frogs test	7.43 ± 4.66	8.68 ± 5.70	50%
Pippo says test	6.33 ± 2.37	6.85 ± 2.15	35%

**^∗^Pre-training, performances at time point T0 for Experimental Condition A and T1 for Experimental Condition B;°Post-training, performances at time point T1 for Experimental Condition A and T2 for Experimental Condition B; ^+^% of children with improve performances, percentage of children with a post-training score at least of 1 point higher than the pre-training.**

The statistical analysis of ER-Lab effects on neuropsychological outcomes in both Experimental Conditions are reported in Table 5. Significant improvements after ER-Lab training were found in Naming and Inhibition speed (*p* = 0.001; *p* = 0.008, respectively) and in Naming Self-correcting responses (*p* = 0.01). No other significant differences emerged in any other delta changes pre- and post-ER-Lab training, neither in visuo-spatial memory and working memory domains, nor in the inhibition of automatic motor responses. No delta change was found during normally academic programs in any neuropsychological measures (*p* > 0.05).

**TABLE 5:** Results of mixed effects model and *post hoc* comparisons on delta changes in all children with SN.

**Neuropsychological outcomes**	**Within baseline effect^+^**	***Post hoc* comparison**	**Training effect^§^**	***Post hoc* comparison**
	
	**Estimated Mean (CI)**	***p***	**Estimated Mean (CI)**	***p***
Forward Corsi Block Tapping test	0.14 (−2.05, 2.33)	0.982	−1.66 (−4.75, 1.43)	0.347
Backward Corsi Block Tapping test	0.53 (−2.35, 3.42)	0.848	−0.60 (−4.63, 3.43)	0.898
Matrices Paths test	4.06 (−5.69, 13.82)	0.499	1.60 (−12.08, 15.29)	0.936
Time in naming condition	−44.08 (−137.86, 49.70)	0.427	−210.08 (−345.21, −74.95)	0.001^∗^
Errors in naming condition	1.48 (−10.53, 13.49)	0.926	−11.31 (−28.49, 5.86)	0.225
Self-correcting responses in naming condition	6.31 (−2.42, 15.03)	0.175	−15.75 (−28.21, −3.29)	0.011^∗^
Time in inhibition condition	−67.39 (−149.39, 14.61)	0.117	−153.50 (−270.62, −36.39)	0.008^∗^
Errors in inhibition condition	2.32 (−22.34, 26.98)	0.959	−33.38 (−68.30, 1.53)	0.063
Self−correcting responses in inhibition condition	−0.28 (−11.24, 10.68)	0.998	−1.90 (−17.42, 13.62)	0.930
Little frogs test	−0.93 (−14.89, 13.03)	0.981	8.43 (−11.58, 28.44)	0.493
Pippo says test	−2.59 (−8.52, 3.33)	0.469	1.76 (−6.61, 10.13)	0.812

**Estimated Mean (CI), the mean and the Confidence of Interval estimated on the basis of the statistical model for each outcome measure. ^+^Within Baseline Effect, differences during normally academic program in both Experimental Conditions, calculated adding delta changes baseline in Experimental Condition B (T1–T0 for Experimental Condition B) and follow-up in Experimental Condition A (T2–T1 for Experimental Condition A); ^§^Training Effect, differences during ER-Lab training in both Experimental Conditions, calculated by adding delta changes for time points T1 and T0 for Experimental Condition A and delta changes for time points T2 and T1 for Experimental Condition B.*^∗^Significant result.*

No difference in the pre-post ER-Lab Delta changes emerged between mild and severe impairment subgroups in any neuropsychological test (*p* > 0.05), while children with attentional problems showed higher pre-post changes in the Simon Says test compared to the subgroup with language problems [*t*(13.56) = 2.39, *p* = 0.032]; no other significant difference emerged in any other neuropsychological outcomes.

**FIGURE 6:**
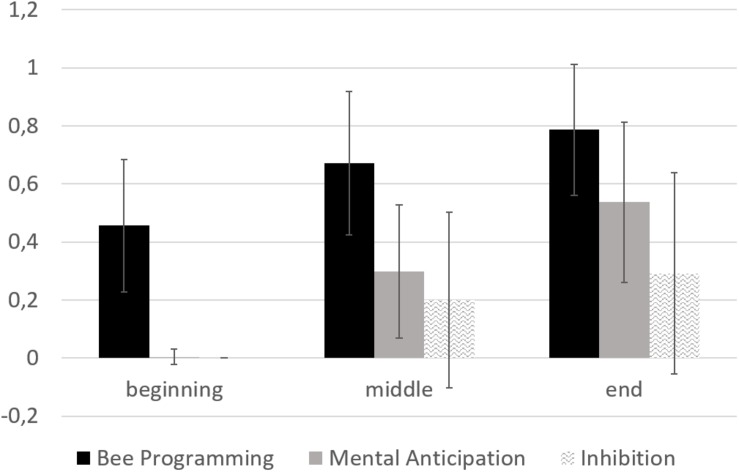
Visual inspection of changing in ER-Lab test at the beginning, middle, and end sessions.

In the ER-Lab test, as shown in Figure 6, the children displayed a positive learning trend on the Bee Programming subscale [*F*(1, 36) = 89.5, *p* < 0.001], with performances significantly higher at the end of ER-Lab training with respect to both the beginning [*t*(36) = −9.5; *p* < 0.001] and middle [*t*(36) = −6.3, *p* < 0.001] sessions. Positive trends were also found on the Mental Anticipation subscale [*F*(1, 35) = 125.8, *p* < 0.001], with significant benefits of training displayed at the end with respect to the beginning [*t*(35) = −11.4, *p* < 0.001] and middle [*t*(36) = −7.7, *p* < 0.001] sessions. As with previous subscales, also on the Inhibition subscale performances were significantly improved during ER-Lab training [*F*(1, 33) = 21.4, *p* < 0.001], being higher at the end in comparison to the beginning [*t*(35) = −5.1, *p* < 0.001] and middle [*t*(34) = −3.9, *p* < 0.001] sessions.

## Discussion

The present study found that ER-Lab training had a significant effect on inhibition skills in a group of children with SN, supporting that it is possible to empower one of the main EFs components in children with SN within an ecological context, incorporating social, emotional, and cognitive significances.

To reach this purpose, the ER-Lab for EFs within schools appeared to be a suitable tool thanks to its technical characteristics, the adaptability and flexibility of interfaces, and its increasing pedagogical implementation (Di Lieto et al., 2017b, 2019). This study was the first attempt to adopt a rigorous and scientific approach, both in terms of study design and of intervention methodology, to improve EFs by ER in a sufficiently large sample of children with SN.

The ER-Lab logbook observations suggested that, first of all, despite the wide variability of clinical problems in the sample, all children showed a high level of interest and motivation during ER activities, and all, except one, performed ER-Lab within small groups of children. According to teachers’ qualitative observations, this setting has been important to favorize social inclusion and more efficient learning. Mutual concrete and verbal feedback among children helped to sustain the gradual development of self-control capacities and careful reflection regarding the pre-set goals to evaluate the need of change or modifications. Moreover, the different methodological and goal adaptations were organized according to the type of neuropsychological or cognitive deficits in order to favorize gradual and efficient learning, following the specific strengths and weaknesses of children with SN. By qualitative observations, methodological and goal adaptations were positively accepted by children, both when they were oriented to the behavior (e.g., breaks or token economy strategies) or to cognitive strategies (e.g., cardkeys or the simplification of robot-programming goals). Not all children with severe motor or intellectual disabilities or with autism accepted the modified Bee-bots, however, because the different shape of Bee-bot may favor self-perception of diversity in comparison with their peers. Nevertheless, children with more severe clinical problems and, thus, with significant difficulties in Bee-bot programming, accepted the modified Bee-bots and used them exclusively or alternatively to the standard Bee-bots.

Concerning the neuropsychological assessment, conducted according to the waitlist randomized trial design, a majority of the children completed all of the tests without relevant difficulties, which is suggestive of the feasibility of a quantitative approach to measure ER-Lab effects in children with SN.

An increasing number of researchers on EF interventions in children with SN employ high-cost technologies, which is not easily accessible or achievable for families or schools (Shinaver et al., 2014). The present study provides a first attempt at implementing an EF intervention in school classes; it is flexible in terms of methodological and goals adaptations for children with SN, taking advantage of the positive characteristics of the new technologies, such as its appeal, the possibility it displays to decompose complex programming into simpler tasks, and the possibility of using ecological, flexible, and low-cost tools.

The main finding of the present study was the significant effect the ER-Lab training had on inhibition skills in terms of speed of processing (Time in Inhibition condition test) and rapid automatization naming in terms of speed of processing and accuracy (Time and Self-correcting responses in Naming condition test). Thus, after the training, children with SN showed a significant increase, in comparison to the pre-training assessment, in the speed of their cognitive control of inappropriate responses and in the number of self-monitoring responses they displayed; this was for the improvement of performances of the Self-correcting responses parameter in the Naming condition test. This result was expected because the ER-Lab activities were implied to inhibit automatic responses through programming activities that trained the capacity to think before acting or to give the opposite response with respect to a certain command (see Table 1 for a more detailed description of activities and cognitive goals). No pre-post differences were found, and this was in contrast to what we expected in relation to our previous study (Di Lieto et al., 2017b), in working memory and in other inhibition tests. It may be hypothesized that, because of the functional heterogeneity of SN, the ER-Lab training may affect mainly inhibition, that is, according to recent literature, the main basic EFs, emerging as single undifferentiated factor in early ages (Wiebe et al., 2008; Fuhs and Day, 2011; Wiebe et al., 2011; Willoughby et al., 2012; Gandolfi et al., 2014). Moreover, although not directly explored in the present study, heterogeneity in the EFs profile in SN, as documented by several studies (Castellanos et al., 2006; Lanfranchi et al., 2010; Kapa and Plante, 2015; Vilgis et al., 2015; Astrea et al., 2016; Margari et al., 2016; Di Lieto et al., 2017a), can also partially explain the smaller ER-Lab training effect in SN than in typical children. In addition, we hypothesized that, as ER-Lab training stressed different abilities, the direct effect on specific EF components, such as inhibition and working memory, may be mild within a heterogeneous population.

Due to the missing data for two clinical subgroups, the small sample size, and the high internal variability of other clinical subgroups, it was not possible to directly compare all the different subgroups; thus, apart from subgroup visual inspections, explorative analyses were conducted on subgroups of children, divided according to the degree of impairment and the neuropsychological problems. The comparisons showed differences in ER-Lab training effects. No difference was found between the two subgroups based on the degree of the impairment in any of the neuropsychological tests, suggesting that the ER-Lab training may have a positive effect in children, both for those with mild difficulties and with a more severe impairment. For that which concerns specific neuropsychological criteria, children with attentional impairment had more benefits in the inhibition of motor responses task when compared to children with language deficit. This specific positive effect of the ER training, therefore, concerns an EF component representing, more than in developmental language disorders, a core deficit in children with attentional problems because it is also associated with a specific neuro-functional pattern (for a meta-analysis see Lei et al., 2015).

The present study has some limitations. First of all, we conducted statistical analyses only in some clinical subgroups based on the type of cognitive or neuropsychological impairments (attention vs. language problems), excluding comparisons with other type of clinical population (cognitive impairment, intellectual deficit, Autism Spectrum disorder, and neuro-motor disabilities) due to missing data for Autism Spectrum Disorder and Intellectual Disability subgroups and due to small sample sizes and heterogeneity of the samples for cognitive impairment and neuro-motor disabilities subgroups. In light of this, the feasibility and efficacy results of this study need to be confirmed in larger samples, differentiated according to neuro-developmental disorders with the addition of other neuropsychological outcome measures to assess children with more severe intellectual and social communication deficits. Despite these limitations, the results of this study seem to be particularly important because they contribute to the implementation of new evidence-based interventions, which may be used in synergy to clinical and home-based trainings in children with SN. Another relevant limitation involves the training transfer effects on school achievements or on school adjustment, that were not investigated and that can be addressed in future studies. Moreover, different tests (for example, to study spatial working memory or other cognitive abilities involved in the training, such as the attention domain) can be utilized in future studies to better understand the ER-Lab effect. Finally, future studies are needed to compare ER training to other training oriented to improve EFs in order to confirm the key points individuated in the literature to define a EFs training as being effective in a clinical sample.

## Conclusion

In conclusion, this study may suggest new and interesting elements about the educational role of robotics in the scholastic system also in children with neurodevelopmental disorders. These activities may favorize both the cognitive learning, exploiting the adaptability of the robots, and the social inclusion thanks to the context of the group setting of the ER activities.

## Data Availability Statement

The datasets generated for this study are available on request to the corresponding author.

## Ethics Statement

The studies involving human participants were reviewed and approved by the Pediatric Ethics Committee of Tuscany Region. Written informed consent to participate in this study was provided by the participants’ legal guardian/next of kin.

## Author Contributions

MD, EC, CP, GS, and EI conceptualized and designed the work, data collection, data analysis, and data interpretation and participated in the writing and critical revision of the manuscript. FC, PD, and GC conceptualized and designed the work and performed the critical revision of the article. All authors approved the final version.

## Conflict of Interest

The authors declare that the research was conducted in the absence of any commercial or financial relationships that could be construed as a potential conflict of interest.
